# Genome Sequences of 14 Siphophages That Infect Serratia marcescens

**DOI:** 10.1128/mra.01212-21

**Published:** 2022-04-12

**Authors:** Emilee L. Carr, McKay E. Wilson, Stephen T. Adams, Daniel K. Arens, Moroni Ayala, Hayden Ayers, Austin Barker, Victoria Beecroft, Emily Bishop, Braden Brundage, Melany J. Carroll, Jacob Chow, Hunter Cobbley, Rhen Davis, Christopher Fajardo, Samuel Flor, David Fuhriman, Rochelle Gaertner Tullis, Austen Gleave, Ciara Green, Tyler Hanis, Trevor Hoggan, Liam Johnson, Jared L. Kruger, Andrew Lambert, Elvira Correa Lazaro, Emily Loertscher, Naomi Marshall, Elise Melhado, Riley Sarabia, Ruchira Sharma, Austin Steffensen, Jared B. Stewart, Tyson Stoker, Andrew Swain, Simeon Toronto, Daniel W. Thompson, J. Zachary Todd, Jamison Walker, Andrew Wilkey, Derrek Wilson, Cynthia L. Hallen, Sherwood R. Casjens, Julianne H. Grose

**Affiliations:** a Department of Microbiology and Molecular Biology, Brigham Young University, Provo, Utah, USA; b Department of Linguistics, Brigham Young University, Provo, Utah, USA; c School of Biological Sciences, University of Utah, Salt Lake City, Utah, USA; d Division of Microbiology and Immunology, Department of Pathology, University of Utah School of Medicine, University of Utah, Salt Lake City, Utah; Loyola University Chicago

## Abstract

We announce the complete genome sequences of 14 *Serratia* bacteriophages isolated from wastewater treatment plants. These phages define two previously undescribed types which we call the Carrot-like phage cluster (phages Carrot, BigDog, LittleDog, Niamh, Opt-148, Opt-169, PhooPhighters, Rovert, Serratianator, Stoker, Swain, and Ulliraptor) and Tlacuache-like phage cluster (Tlacuache and Opt-155).

## ANNOUNCEMENT

Abundant in the environment, Serratia marcescens is an opportunistic pathogen that frequently causes hospital-acquired infections, particularly catheter-associated bacteremia and urinary tract infections, as well as wound infections. Antibiotic-resistant strains are common, making phage therapy a possible alternative treatment (1, 2). The isolation, complete genome sequences, and annotation of 14 *Serratia* siphophages are presented.

The 14 bacteriophages were isolated from wastewater treatment plants in the western United States (Table 1). Briefly, LB-based enrichment cultures using 0.5 mL of overnight S. marcescens HY 150 (ATCC 27143) culture, 0.5 mL sewage, and 4 mL LB were incubated at 37°C for 48 to 72 h. Bacteria were pelleted by centrifugation, and the 50 μL supernatant was incubated with 0.5 mL bacterial overnight culture and plated with LB top agar for single plaques that were picked, and this single plaque isolation was repeated at least three times. Lysates (>10^8^ PFU/mL) were made by incubating a final plaque with bacterial overnight (0.5 mL) culture in ∼4 mL LB (37°C for 48 to 72 h) prior to centrifugation. Genomic DNA was isolated with the Norgen Biotek phage DNA isolation kit (Canada) and prepared for paired-end Illumina sequencing with either the New England Biolabs (NEB) Ultra II DNA kit followed by 150-bp sequencing on an iSeq instrument (Niamh, Serratianator, and Ulliraptor), the Illumina TruSeq DNA Nano kit followed by 250-bp sequencing on the HiSeq 2500 instrument (Opt-148, BigDog, LittleDog, Stoker, Swain, PhooPhighters, Opt-155, and Tlacuache), or 150-bp sequencing on the MiSeq instrument (Carrot, Rovert, and Opt-169). Trimmed contigs were assembled using the preset *de novo* assembly of Geneious v.R11 for HiSeq and MiSeq data or v.8.0.5 for iSeq data (3) and subsequently annotated using DNA Master v.5.0.2 (4) and GeneMarkS (5). All software was used at default settings. All 14 phages were determined to have long noncontractile tails and thus be siphophages by negative-stain electron microscopy at the BYU microscopy center.

**TABLE 1:** Sequencing summary and basic properties of 14 *Serratia* siphophages

Phage name^*a*^	GenBank accession no.	SRA accession no.	Total no. of reads	Fold coverage range (×) (mean)	Length (bp)	GC content (%)	Sewage sample GPS (N, W)
vB_SmaS_Carrot	OL539439	SRR17231348	19,919	1–179 (125.7)	41,293	45.7	33.7392, 104.9903
vB_SmaS_Niamh	OL539455	SRR17231360	101,716	216–578 (364.7)	42,053	46	40.2338, 111.6585
vB_SmaS_Ulliraptor	OL539442	SRR17231373	44,691	65–668 (441.2)	42,052	46	41.1324, 111.9302
vB_SmaS_Serratianator	MW021755	SRR17231370	123,270	65–668 (441.2)	42,052	46	41.1324, 111.9302
vB_SmaS_Stoker	OL539464	SRR10580541	103,361	15–1,393 (614.6)	41,797	46.4	33.4152, 111.8315
vB_SmaS_Littledog	OL539456	SRR10580537	3,380	1–38 (17)	41,738	45.8	^*b*^
vB_SmaS_Opt-148	MW021766	SRR10580536	267,924	222–3,240 (1,426.8)	41,293	45.7	^*b*^
vB_SmaS_Bigdog	MW021763	SRR10580535	780,873	757–12,056 (4,248.1)	42,495	45.7	^*b*^
vB_SmaS_Swain	OL539438	SRR10580534	270,531	125–4,919 (1,591)	41,292	45.7	33.4274, 117.6126
vB_SmaS_Rovert	MW021761	SRR10580538	242,717	40–6,693 (1,505.7)	38,613	42.3	40.1150, 111.6549
vB_SmaS_Opt-169	MW021767	SRR10580543	6,547	8–1,694 (846.3)	38,609	42.3	33.1959, 117.3795
vB_SmaS_PhooPhighters	OL539441	SRR10580539	239,567	15–1,393 (614.6)	39,188	42.2	40.1652, 111.6108
**vB_SmaS_Opt-155**	OL539452	SRR10580544	1,780,701	7,572–22,560 (10,288.5)	42,792	51.9	33.9806, 117.3755
**vB_SmaS_Tlacuache**	OK499995	SRR10580545	429,314	1,284–4,016 (2,255)	42,679	51.6	33.7392, 104.9903

a The Tlacuache cluster phages are indicated by bold type, all other phages belong to the Carrot cluster.

b These phages were isolated from unrecorded sewage from the western United States.

Our published strategy of requiring homology over >50% of the genome for phage “cluster” membership (6) places these phages in two distinct and well-defined clusters (approximately International Committee on Taxonomy of Viruses [ICTV] families) by dot plot comparison. With the previously reported *Dickeya* phage Sucellus (GenBank accession no. MH059634), 12 of these phages compose a novel Carrot-like *Enterobacteriales* phage cluster that contains the following three subclusters (approximately ICTV genera) of highly related phages that have >90% nucleotide sequence identity over the length of the genome (7, 8): subcluster A—Carrot, BigDog, LittleDog, Niamh, Serratianator, Swain, Stoker, Ulliraptor, Opt-148; subcluster B—Rovert, Opt-169, PhooPhighters; and subcluster C—Sucellus. The remaining two phages, Tlacuache and Opt-155, along with the previously reported *Serratia* phage Serbin (GenBank accession no. MK608336 [8]), share ≥92% nucleotide identity as determined by BLASTN and define a second novel *Enterobacteriales* phage cluster.

These Carrot-like and Tlacuache-like phages have different GC contents, averaging 45% and 52%, respectively, whereas the S. marcescens GC content is ∼59%. All 14 bacteriophage genomes circularized upon assembly, except for LittleDog and BigDog. Analysis of raw sequencing reads with PhageTerm (7) suggests the Carrot-like genomes have cohesive ends, whereas Tlacuache-like genomes likely utilize a headful DNA packaging strategy.

### Data availability.

The accession numbers for all 14 bacteriophages can be found in Table 1.
